# Longitudinal Analysis of the Intestinal Microbiota in the Obese Mangalica Pig Reveals Alterations in Bacteria and Bacteriophage Populations Associated With Changes in Body Composition and Diet

**DOI:** 10.3389/fcimb.2021.698657

**Published:** 2021-10-19

**Authors:** Haley A. Hallowell, Keah V. Higgins, Morgan Roberts, Robert M. Johnson, Jenna Bayne, Herris Stevens Maxwell, Terry Brandebourg, Elizabeth Hiltbold Schwartz

**Affiliations:** ^1^ Department of Biological Sciences, Auburn University, College of Science and Mathematics, Auburn, AL, United States; ^2^ Department of Animal Sciences, College of Agriculture, Auburn University, Auburn, AL, United States; ^3^ Department of Clinical Sciences, College of Veterinary Medicine, Auburn University, Auburn, AL, United States

**Keywords:** microbiota, obesity, swine, diet, age, Mangalica

## Abstract

Due to its immunomodulatory potential, the intestinal microbiota has been implicated as a contributing factor in the development of the meta-inflammatory state that drives obesity-associated insulin resistance and type 2 diabetes. A better understanding of this link would facilitate the development of targeted treatments and therapies to treat the metabolic complications of obesity. To this end, we validated and utilized a novel swine model of obesity, the Mangalica pig, to characterize changes in the gut microbiota during the development of an obese phenotype, and in response to dietary differences. In the first study, we characterized the metabolic phenotype and gut microbiota in lean and obese adult Mangalica pigs. Obese or lean groups were created by allowing either *ad libitum* (obese) or restricted (lean) access to a standard diet for 54 weeks. Mature obese pigs were significantly heavier and exhibited 170% greater subcutaneous adipose tissue mass, with no differences in muscle mass compared to their lean counterparts. Obese pigs displayed impaired glucose tolerance and hyperinsulinemia following oral glucose challenge, indicating that a metabolic phenotype also manifested with changes in body composition. Consistent with observations in human obesity, the gut microbiota of obese pigs displayed altered bacterial composition. In the second study, we characterized the longitudinal changes in the gut microbiota in response to diet and aging in growing Mangalica pigs that were either limit fed a standard diet, allowed *ad libitum* access to a standard diet, or allowed *ad libitum* access to a high fat-supplemented diet over an 18-week period. As expected, weight gain was highest in pigs fed the high fat diet compared to *ad libitum* and limit fed groups. Furthermore, the *ad libitum* and high fat groups displayed significantly greater adiposity consistent with the development of obesity relative to the limit fed pigs. The intestinal microbiota was generally resilient to differences in dietary intake (limit fed *vs ad libitum*), though changes in the microbiota of pigs fed the high fat diet mirrored changes observed in mature obese pigs during the first study. This is consistent with the link observed between the microbiota and adiposity. In contrast to intestinal bacterial populations, bacteriophage populations within the gut microbiota responded rapidly to differences in diet, with significant compositional changes in bacteriophage genera observed between the dietary treatment groups as pigs aged. These studies are the first to describe the development of the intestinal microbiota in the Mangalica pig, and are the first to provide evidence that changes in body composition and dietary conditions are associated with changes in the microbiome of this novel porcine model of obesity.

## Introduction

The prevalence of obesity in adult populations is approaching pandemic levels. For instance, currently more than 650 million adults worldwide and greater than 40% of adults in the United States are considered obese (CDC, 2020) This poses a serious public health crisis as obesity is associated with multiple comorbidities including metabolic disease, cardiovascular disease, and gastrointestinal diseases (Obesity and overweight). While the etiology of obesity is not fully understood, it is clear that a chronic dysregulation of energy balance drives expansion of adipose tissue, and this associates with the development of meta-inflammation that in turn promotes impaired insulin sensitivity and ultimately the myriad of downstream comorbidities (Li et al., 2018). Due to the immunomodulatory and energy-harvesting potential of the intestinal microbiota, it has been implicated as a contributing factor in the development of metainflammation. Further, obesity-associated changes in the intestinal microbiota have also been linked to the promotion of the overnutrition underlying the expansion of adipose tissue (Turnbaugh et al., 2009; Ley, 2010; Ridaura et al., 2013; Duvallet et al., 2017; González-Muniesa et al., 2017; Gomes et al., 2018; Yang et al., 2021). A better understanding of these links could allow the development of targeted therapies to either prevent the onset of obesity or to uncouple obesity from downstream disease states.

The mammalian gastrointestinal tract is home to a large collection of microorganisms collectively known as the gut microbiota. All domains of life are represented within this diverse and dynamic microbial ecosystem, with a majority of constituents being bacteria and viruses, namely bacteriophage. (Turnbaugh et al., 2009; Ley, 2010; Wang et al., 2019a; Chevallereau et al., 2021). This bacterial component is dominated by 4 major bacterial phyla (in order of dominance): Bacteroidetes, Firmicutes, Protetobacteria, and Actinobacteria (Turnbaugh et al., 2009; Ley, 2010; Duvallet et al., 2017; Wang et al., 2019a). Investigations of the intestinal bacteriome have revealed a metabolically active microbial community that has many bidirectional interactions with the mammalian host (Mehal, 2013; Ridaura et al., 2013; Wang et al., 2019a). For example, diets high in plant polysaccharides have been directly linked to an increase of bacterial species belonging to the phylum Bacteroidetes. More specifically, *Prevotella* spp. have a strong connection to diets high in fiber due to their ability to metabolize complex carbohydrates (de Filippo et al., 2010; Wu et al., 2011; David et al., 2013). Interestingly, *Bacteroides* spp. tend to decrease in response to the same diet, and be more tightly linked to a diet rich in animal products (de Filippo et al., 2010; Wu et al., 2011; Flint et al., 2012; Jandhyala et al., 2015). However, bacteria do not exist in isolation within the microbiome; their predators, bacteriophage, often exist in similar or greater abundance than their bacterial host (Chevallereau et al., 2021). The impact of bacteriophage on the form and function of the gut microbial ecosystem has only just begun to be appreciated.

Other features of the mammalian host can contribute to the composition and proportion of microbial symbionts within the gut microbiota, such as the host’s environment, disease state, antibiotic usage, and diet (Rinninella et al., 2019). Among these, the impact of diet on the microbiome has been the most thoroughly examined. There is now a large body of literature examining the interplay between diet, the microbiome, and host health. However, studies have often reported conflicting results. For example, a recent meta-analysis found no correlation between specific bacterial taxa and bacterial richness with diet-induced obesity (Duvallet et al., 2017). Further, even less is known about how bacteriophage populations are impacted by an obesogenic state (Schulfer et al., 2020). Establishment of novel models that facilitate the study of these dynamics should promote an enhanced understanding of mechanisms linking the microbiota to the health status of the host.

To date, most published studies defining the composition and functionality of the gut microbiota in human health and obesity have utilized mouse models. However, pigs have been proposed as a more accurate model for human obesity, as more similarities are seen between pigs and humans in terms of anatomy, physiology, and nutrient digestion (Litten-Brown - et al., 2010; Heinritz et al., 2013). Both pigs and humans are omnivores and lack anatomically discreet depots of brown fat within the vasculature. Additionally, the proportion of skeletal muscle and adipose tissue to total body mass and circulating levels of glucose are very similar between pigs and humans (Litten-Brown - et al., 2010; Heinritz et al., 2013). Furthermore, given the well-developed literature concerning the use of swine as a biomedical model to study atherosclerosis, cardiovascular disease, and diabetes, pigs are well-positioned as an experimental solution to overcome limitations inherent in using rodent models to study metabolic syndrome and obesity (Zou et al., 2019; Karimi et al., 2020).

While multiple porcine models for obesity currently exist, each have some limitations. To date, no swine model spontaneously displays the full spectrum of metabolic dysregulation associated with human obesity. Also, few swine models of hyperphagic obesity currently exist (Pedersen et al., 2013; Spring et al., 2020). In this regard, the fatty Mangalica pig, capable of achieving a body composition comprised of 70% adipose tissue by mass, represents a novel alternative (Molnár et al., 2014). A voluntary chronic overnutrition drives this extreme, early onset, morbidly obese phenotype that associates with a spontaneous proinflammatory, insulin resistant metabolic phenotype in these pigs as they age (Roberts, et al, under review). Despite the Mangalica displaying great potential to serve as a relevant animal model of obesity and its complications, neither the normal development of non diet-induced effects upon its gut microbiota have been characterized.

Because the gut microbiota of this novel obese porcine model has never been explored, we characterized the metabolic phenotype and gut microbiota in adult lean and obese Mangalica pigs. Further, we then characterized the longitudinal changes in the gut microbiota over an 18-week period in response to diet and aging utilizing juvenile Mangalica pigs that were either given restricted access to a standard diet (lean), allowed *ad libitum* access to a standard diet (obese), or allowed *ad libitum* access to a high fat-supplemented diet (diet-induced obese). These studies are the first to describe the overall development of the intestinal microbiota in the Mangalica pig, and represent a key first step toward the development of this breed as a useful model to study mechanisms linking the intestinal microbiota to the development of obesity and downstream metabolic states.

## Materials And Methods

### Animals, Diets, and Fecal Sample Collection

Purebred Mangalica pigs were obtained from the Auburn University research herd housed at the Auburn University Swine Research and Education Center. In the first study, ten weaned pigs were individually housed in pens and provided *ad libitum* access to water. Voluntary feed intake was determined daily by weighing back orts, and body weights were determined weekly. To establish our limit fed (lean) and *ad libitum* (obese) groups, five pigs were allowed *ad libitum* access to the balanced basal diet (**Table 1**) while the remaining five pigs were fed the basal diet at levels that were 40% of the voluntary intake of their *ad libitum* fed counterparts for the previous day. Despite feed restriction, daily rations for all pigs exceeded the nutrient recommendations for healthy growth of the breed (Council, 2012). Body composition was assessed *via* ultrasound at 26 and 52 weeks on trial. Pigs underwent oral glucose tolerance tests to assess glycaemia and insulinemia. Fecal samples were collected aseptically from post-pubertal pigs once clinical parameters of obesity were observed within the *ad libitum* group. For the second (longitudinal) study, twelve post-weaned pigs were housed two per pen. Pigs penned together received the same dietary treatment. Limit fed and *ad libitum* groups were established by utilizing the same dietary strategy as described above. The *ad libitum* + high fat (HF) group was established by allowing *ad libitum* access to the basal diet supplemented with 28% dietary fat (**Table 1**). All pigs were provided *ad libitum* access to water. Fecal samples were collected aseptically one week prior to the assignment of pigs to their respective dietary treatments and then one-, ten-, fourteen-, and eighteen weeks following their transition to the respective diets. Body weights were recorded weekly and body composition was assessed *via* ultrasound bi-weekly while pigs were on trial.

**Table 1:** Formulation and composition of experimental diets (as-fed basis).

Item	Diet	
	Control	High Fat
Ingredient, g/kg		
Corn	727.00	577.00
Soybean meal, 47.5% CP	107.00	86.00
Dried Distillers Grains^1^	100.00	80.00
Dicalcium Phosphate	0.16	0.13
Limestone	11.51	9.78
Salt	4.00	3.25
Vitamin-trace mineral premix	0.45	0.45
Soybean oil	46.00	250.00
Calculated composition		
ME^1^, mcal/kg	3.47	4.75
Crude protein, %	13.80	10.90
Fat, %	4.60	25.00
Ca, %	.68	.54
Available P, %	.45	.36

^1^Metabolizable energy.

### Ultrasound

Real-time ultrasound was performed on all pigs to assess body composition in growing animals by determining on test ultrasound 10^th^ rib subcutaneous fat depth and Longissimus muscle depth according to Perkins et al. (2014). All ultrasound data was collected by the same Ultrasound Guidelines Council certified technician using an Aloka 500 (Aloka America, Wallingford, CT) with a 17 cm transducer using CUP Lab image capture software.

### Oral Glucose Tolerance Test and Insulin Measurements

Lean and obese pigs from the first study were subjected to an OGTT when obese pigs reached an average body weight of 160 kg. Pigs were fitted with jugular catheters and allowed to recover for 7 days. Catheters were flushed with heparinized saline twice daily to maintain patency. During the OGTT, pigs were fasted for 24 h and then offered a control diet equal to 1% of their body weight that had been supplemented with glucose equivalent to 2 g per kg BW. Blood was obtained 15 minutes before and 15, 30, 60, 120, and 180 min after consumption of the glucose dose. Blood was directly analyzed for glucose using a clinical glucose analyzer (YSI 2300 STAT Plus, YSI Inc., Yellow Springs, OH). To facilitate insulin measurement, blood samples were centrifuged (3000 x g, 10 min, 4°C) and resulting plasma was collected and stored at -80°C until analysis. Plasma insulin (porcine insulin ELISA kit, ALPCO, Salem, NH) was determined using commercially available kits according to manufacturer instructions. Glucose (mg/dl) and insulin (µU/ml) data are presented as area under the curve (AUC).

### Shotgun Metagenomic Sequencing of the Gut Microbiota

Immediately after collection, DNA was extracted from fecal samples using the E.Z.N.A kit (Omega). DNA samples were sent to Hudson Alpha Genome Sequencing Center (Huntsville, Al.) for shotgun metagenomic sequencing. Sequencing was carried out using an Illumina HiSeq 2500 v4 with a 2 x 125 paired-end sequencing 200 million reads. For the preliminary study, each sample was sequenced individually, while in the longitudinal study, DNA samples were pooled by pen (2 pigs per sample). For annotation of samples, an in-house annotation pipeline was used. The metagenomic pipeline can be found at: https://github.com/haleyhallowell/metagenome-annotation-pipeline/blob/main/annotation.sh. Briefly, quality was assessed using FastQC (Babraham Bioinformatics). Using Trimmomatic, sequencing adapters and low-quality sequences (Q-score < 30) were removed (Bolger et al., 2014). Host sequences were then removed using BWA by mapping reads to the host genome (*Sus scrofa* NCBI v10.2) (Li, 2013). Reads were then assembled using the Iterative De Brujin Assembler (IDBA-UD), and reads were mapped back to the assembly using Bowtie2 (Langmead and Salzberg, 2012; Yu et al., 2012). Mapped reads were then annotated using MetaPhlAn3 (Beghini et al., 2021).

### Statistics

For statistical analysis in the first study, growth and clinical characteristics were analyzed as a completely randomized block design using a mixed linear model of SAS v9.2 with individual animal serving as the experimental unit, i.e., individual block (SAS Institute, Inc., Cary, NC). In the second (longitudinal) study, weights and back fat measurements were analyzed using a one-way ANOVA with a Tukey’s *post-hoc* test. This was performed in the R-studio platform.

Microbiome analysis was performed using outputs generated through MetaPhlAn3; this included both relative abundance and raw hit counts. To determine the alpha diversity between treatment groups in both sets of samples, raw hit counts were used to generate a Bray-Curtis matrix, and plotted using non-metric multidimensional scaling (*nMDS*) using the vegan package in R (Oksanen et al., 2020). Significant differences between different treatment groups, as well as interactions between covariates, were determined using a PERMANOVA, by employing the adonis function in the vegan package (Jari Oksanen). Stacked bar plots displaying relative abundance of the datasets were generated in the phyloseq (McMurdie and Holmes, 2013). To detect differentially abundant taxa between our treatment groups, a differential abundance analysis was performed using DeSeq2 (Love et al., 2014). Briefly, raw hit counts were rlog transformed, and a Wald’s test was used to determine significantly different taxa. Adjusted p-values (q-values) were then generated using the Benajmini-Hochberg (FDR) correction to account for false positives (Benjamini and Hochberg, 1995). Pearson’ correlation coefficients were calculated using the package *psych* (Revelle, 2021). Pearson correlation plots were generated in R studio using the package *ggcorplot* (Kassambara, 2019), only including relationships with a correlation coeffect greater than |0.6|.

## Results

### Mature Lean (Restricted) and Obese (Ad Libitum) Mangalica Pigs Exhibit Divergent Body Composition and Metabolic Phenotypes

Our first study was conducted to characterize the intestinal microbiota of mature lean and obese Mangalica pigs. Initial body weights of the juvenile pigs were not different between the limit fed and *ad libitum* groups (p > 0.92). As expected, the final body weights of mature *ad libitum* pigs weighed 53% more than their limit fed counterparts (p < 0.001; **Table 2**). Subcutaneous fat depth was 70% greater in *ad libitum vs*. limit fed pigs (p < 0.001) while muscle depth between the two groups was not significantly different (p > 0.73) suggesting differences in body weight were reflective of differences in adiposity rather than skeletal muscle mass (**Table 2**).

**Table 2:** Body composition of initial cohort of adult pigs demonstrating lean and obese phenotypes^1^.

Variable	Limit Fed	Ad Libitum	P-value
Number of pigs	5	5	NA^2^
Body weight, kg	110.1 ± 1.9	167.8 ± 4.9	0.001
Subcutaneous fat, mm	36.1 ± 3.2	61.0 ± 5.2	0.001
Longissimus dorsi, mm	47.3 ± 1.3	50.5 ± 2.1	0.73

^1^Values are means ± standard errors.

^2^NA, not applicable.

**Figure 1:**
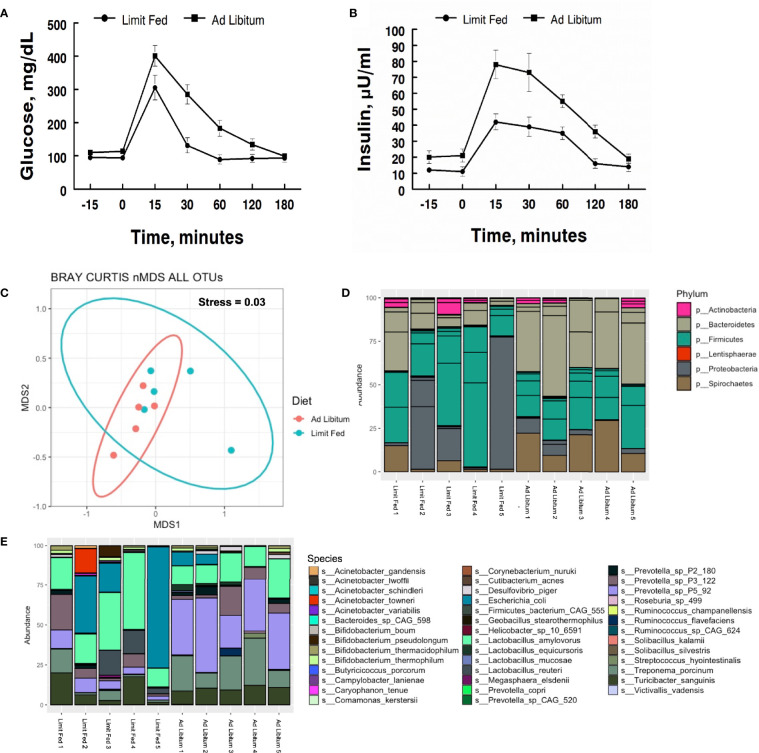
Characterization of metabolic parameters and intestinal microbiota in *ad libitum* and limit fed Mangalica pigs. Oral glucose tolerance test (OGTT) conducted on lean and obese pigs. **(A)** Plasma glucose and **(B)** insulin levels were measured following administration of an oral dose of glucose (2 g/kg BW) to fasted pigs. **(C)** nMDS ordination plot was generated using a Bray-Curtis dissimilarity index. The stress associated with this ordination is 0.003. Bacterial **(D)** Phylum, and **(E)** Species level, composition in Mangalica pigs on either a limit fed or *ad libitum* feeding. Differential abundance statistical results outlined in **Supplementary Tables 1**, **2**.

To determine the impact of adiposity on blood glucose and insulin levels in the mature limit fed and *ad libitum* pigs, an oral glucose tolerance test (OGTT) was performed. Fasting glucose levels were significantly higher in *ad libitum versus* limit fed pigs at time -15 min (p < 0.05; **Figure 1A**). In response to glucose administration, a significant increase in blood glucose levels was observed above baseline by 15 minutes in limit fed and *ad libitum* pigs, with values being significantly higher in *ad libitum versus* limit fed pigs at the peak of the curves. Furthermore, blood glucose values returned to baseline levels by 30 minutes in limit fed pigs after the initial dose, while glucose remained elevated in *ad libitum* pigs, with values not returning to baseline levels until 180 minutes post dosing. Overall, the glucose AUC was increased by 42% for the *ad libitum* Mangalica pigs compared to limit fed counterparts (p < 0.001; **Figure 1A** and **Table 3**). Consistent with the glucose data, fasting insulin levels were significantly higher in *ad libitum versus* limit fed pigs (p < 0.05; **Figure 1B**). Like the glucose response, plasma insulin levels rose significantly from baseline by 15 minutes in both limit fed and *ad libitum* pigs, with peak insulin values being almost twice as high in *ad libitum versus* limit fed pigs. Insulin values returned to baseline levels by 120 minutes in both limit fed and *ad libitum* pigs. Overall, the insulin AUC was increased 79% for *ad libitum* Mangalica pigs compared to limit fed counterparts (p < 0.001; **Figure 1B** and **Table 3**). Several measures of insulin sensitivity were then utilized to assess plasma glucose and insulin values from an oral glucose tolerance test (OGTT) to determine if the *ad libitum* pigs developed insulin resistance (**Table 3**). Utilizing fasted values, HOMO and QUICKI indexes both indicated that pigs fed an *ad libitum* diet displayed impaired insulin sensitivity (**Table 3**). Using peak curve values compared to fasted baseline, the Masuda index likewise indicating an insulin resistant state in *ad libitum* fed pigs compared to their limit fed counterparts (**Table 3**). As expected, the mature *ad libitum* cohort developed many of the phenotypic and metabolic hallmarks of obesity in humans.

**Table 3:** Effect of adiposity on indexes of insulin sensitivity during OGTT challenge in adult lean and obese pigs^1^.

Variable	Limit Fed	Ad Libitum	P-value
Number of pigs	5	5	NA^2^
Glucose AUC^3^	23,400 ± 1,910	33,280 ± 2,140	0.001
Insulin AUC^3^	5,010 ± 599	8,955 ± 674	0.001
QUICKI^4^	.35	.28	0.05
HOMA-IR^5^	1.50	6.25	0.001
HOMA-B^6^	149	122	0.05
HOMA-S^7^	60	36	0.01
Matsuda Index^8^	4.54	1.70	0.001
Insulinogenic Index^9^	.46	.20	0.001
Disposition Index^10^	2.1	.34	0.001

^1^Values are means ± standard errors or simple means.

^2^NA, not applicable.

^3^AUC, area under the curve.

^4^QUICKI, Quantitative Insulin Sensitivity Check Index Visual; normal ranges between 3-.45 and insulin resistance is <.3; lower numbers reflect greater insulin resistance.

^5^HOMA-IR, Homeostatic model assessment- insulin resistant; normal is indicated by values lower than ≤ 1.8.

^6^HOMA-B, Homeostatic model assessment- insulin resistant-β-cell function.

^7^HOMA-S, Homeostatic model assessment- insulin resistant-insulin sensitivity.

^8^Matsuda Index, whole body insulin resistance is indicated for values ≤ 2.5.

^9^Insulinogenic Index, defects in insulin secretion are indicated for values < 0.4.

^10^Disposition Index, (Insulinogenic index)*(Matsuda index); normal is indicated for values < 1.

### Shotgun Metagenomic Analysis of Fecal Microbiota Demonstrate Altered Microbial Composition in Ad Libitum Fed Pigs *vs*. Limit Fed

To characterize the intestinal microbiota of mature limit fed and *ad libitum* pigs, shotgun metagenomic analysis was conducted on fecal samples. Rarefaction curves were generated to assess sequencing depth. Each sample reached a plateau, indicating the presence of more sequences than OTUs, signifying adequate sequencing depth was achieved (**Supplementary Figure 1**). To assess the microbiota of our limit fed and *ad libitum* cohorts holistically, we performed non-metric dimensional scaling (nMDS) using a Bray-Curtis dissimilarity matrix to determine the degree of dissimilarity between each sample. The stress, or “fit” of the model was 0.03, which is within the acceptable range (fit < 0.3) indicating that this is an appropriate representation of the dissimilarity of each sample in 2D space. Most individuals clustered with their cohort, indicating congruency between cohort members. Although the clusters were distinct, limit fed and *ad libitum* microbiota samples plotted close to each other (**Figure 1C**). Diet did have a significant influence on the dissimilarity of our treatment groups (PERMANOVA, p*=* 0.012, R^2^ = 0.37182). Thus, the limit fed and *ad libitum* cohorts had a somewhat similar gut microbiota bacterial consortia, likely due to exposure to identical feed and adjacent housing. However, the clustering seen between these groups points to the *amount* of feed received as being a significant driving factor in the structuring of the microbiota.

Next, we wanted to determine the compositional changes that were driving the dissimilarity between our two cohorts. To do this, we calculated the relative abundance at the taxonomic level of bacterial phyla and species (**Figure 1D**). The major bacterial populations present within the Mangalica intestinal microbiota were consistent with the well-documented, healthy consortia of microorganisms reported in the literature in multiple models, such as humans, mice, and pigs (Turnbaugh et al., 2009; Wang et al., 2019a; Spring et al., 2020). The limit fed cohort’s intestinal microbiota was dominated by 4 main phyla: Firmicutes, Bacteroidetes, Proteobacteria, and Spirochaetes. (**Figure 1B** and **Supplementary Table 1**). We observed a higher abundance of Spirochaetes (p= 0.02) and Bacteroidetes, and a lower abundance of Proteobacteria (p= 0.0009) and Firmicutes (p= 0.016) in our *ad libitum* fed pigs. To further resolve these differences, we evaluated bacterial composition at the level of species in our 2 cohorts. Though there was variation from animal to animal, the limit fed cohort was dominated by *Lactobacillus amylovorus*, *Escherichia coli*, *Treponema porcinum*, *Turicibacter sanguinis*, and multiple *Prevotella* spp. such as *Prevotella sp P3-122* and *Prevotella sp P5-92* (**Figure 1E** and **Supplementary Table 1**). The *ad libitum* cohort harbored similar species, however we observed an increase in *Treponema porcinum*, *Prevotella sp P5-92* and *Bifidobacterium boum* (p= 1.16E -14) and a decrease in *Lactobacillus amylovorus*, and *Lactobacillus reuteri* (p= 0.02) (**Figure 1E**). To our knowledge, this is the first report to define the microbial populations within the Mangalica gut microbiome. Additional taxonomic classification at the level of family and genus is available in **Supplementary Figure 2**. Data presented in these additional figures agree with and support the description of the microbiome given above.

An advantage of our shotgun metagenomics approach was the ability to monitor all constituents of the gut microbiota, particularly bacteriophage. Given that these viruses prey on bacteria, they represent a key selective force in regulating the bacterial composition of the microbiome (Howe et al., 2016; Gogokhia et al., 2019; Hsu et al., 2019; Lin et al., 2019). Overall, individual variability was much greater within bacteriophage populations as compared to bacterial populations in this cohort. Given the highly variable nature of our samples, especially at the taxonomic level of species, we were not able to detect any meaningful shifts within bacteriophage populations at this final endpoint of obesity (**Supplementary Figure 3** and **Supplementary Table 2**).

Taken together, we observed significant shifts in the intestinal microbiota in the natural feeding model of the Mangalica pig. These disruptions within the *ad libitum* group’s gut microbiota were seen in parallel with the phenotypic and metabolic symptoms indicating that like obesity in humans, obesity-associated conditions are associated with shifts in microbial populations within the gut.

### Juvenile Mangalica Pigs Exhibit Different Body Compositions When Fed Divergent Diets During a Longitudinal Analysis Across 18 Weeks

**Figure 2:**
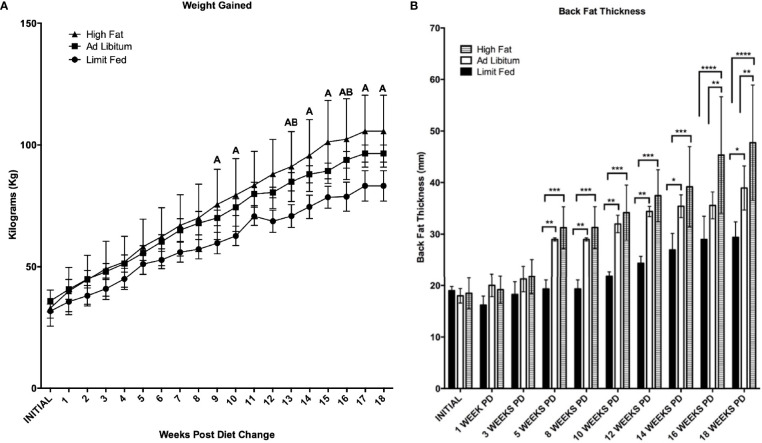
Body weight **(A)** and Back Fat **(B)** change over time. Weekly weight change and biweekly backfat change over time is shown for the limit fed, *ad libitum*, and the *ad libitum* + high fat groups. Group differences over the course of dietary treatment were analyzed by ANOVA. All data points are shown as group mean ± SD. For **(A)**, Tukey’s *post hoc* test results are described as A (represents a *p* < 0.05 for *ad libitum* + high fat compared to limit fed) or B (represents p < 0.05 for *ad libitum* compared to limit fed groups). For **(B)**, *p < 0.05, **p < 0.01, ***p < 0.001, ****p < 0.0001).

As expected of growing pigs, all groups increased in body weight over the 18 weeks of the experiment (**Figure 2A**). However, the *ad libitum* (AL) and high fat (HF) piglets gained weight rapidly compared to limit fed (LF) counterparts and body weights between the three cohorts significantly diverged starting at 9 weeks on the diet (p < 0.05). Further, animals on the high fat diet gained significantly more weight than both the *ad libitum* and limit fed groups starting at 13 weeks (p < 0.05). Both the *ad libitum* and high fat fed groups exhibited significantly greater adiposity compared to the limit fed piglets beginning at 5 weeks on the diet (p < 0.01, p < 0.001, respectively) (**Figure 2B**). High fat fed piglets exhibited significantly greater adiposity than the *ad libitum* group starting at 16 weeks (p < 0.01). In contrast, the limit fed animals showed no significant increase in back fat accumulation over the course of the experiment (**Figure 2B**). These data indicate that diet-induced differences in adiposity were achieved between dietary treatments.

### Mangalica Pigs Exhibit Progressive Changes in the Gut Microbiota Influenced by Age and Diet

**Figure 3:**
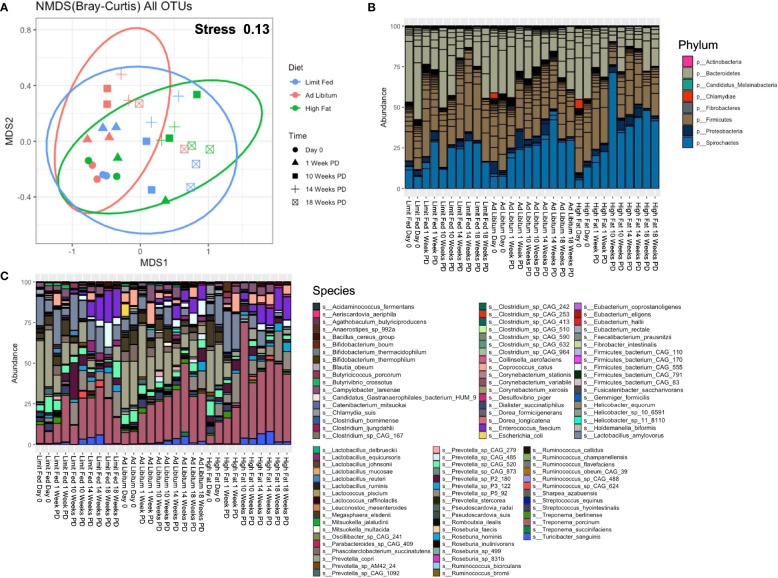
Longitudinal evaluation of bacterial composition following dietary exposure in weaned Mangalica Pigs. **(A)** An nMDS ordination plot was generated using a Bray-Curtis dissimilarity matrix. The shapes represents the time point in which the pigs were sampled, while the color represents the diet administered. The stress for this plot was 0.134. **(B)** Phylum, **(C)** and Species level bacterial composition in Mangalica pigs fed either a limit fed, *ad libitum*, or *ad libitum* + high fat diet prior to and after 1, 10, 14, and 18 weeks of dietary exposure. Differential abundance statistical results are outlined in **Supplementary Tables 3**, **4**.

We next wanted to determine how the microbiota composition changed over time in response to our respective diets. To do this, fresh-catch fecal samples were collected at 1 Week, 10 Weeks, 14 Weeks and 18 Weeks post-dietary exposure (PD), and shotgun metagenomic sequencing was performed. Sequencing depth was assessed through rarefaction curves (**Supplementary Figure 4**). Each sample reached a plateau, confirming adequate sequencing depth. To first assess overall differences in the microbiomes between our diet groups, we again performed non-metric dimensional scaling (nMDS) using a Bray-Curtis dissimilarity matrix (**Figure 3A**). We obtained a stress of 0.13, indicating that our model was a good representation of the dissimilarity between samples. Both diet and time had a significant effect on the differences between group (p= 0.003; p= 0.0010), with diet explaining 15% of the variation and time explaining 41% of the variation. Additionally, there was no interaction between time and diet (p= 0.201) It’s important to note that the diets were fundamentally the same (only different in quantity), with the exception of the high fat group which were given an additional component of high dietary fat. In addition, it is known that the intestinal microbiome responds to aging of the host (Jurburg and Bossers, 2021). Consistent with this, both diet and time were major drivers of dissimilarity in our system.

Having assessed overall differences between the groups throughout the experiment, our next goal was to determine how the composition of the gut microbiota corresponded to dissimilarity. We also sought to compare the microbial composition from this longitudinal study with the results of the initial study (**Figure 1**). We observed similar overall microbial profiles as in our initial study utilizing limit fed and *ad libitum* cohorts. The intestinal microbiota of all groups was dominated by 4 major phyla: Firmicutes, Bacteroidetes, Proteobacteria and Spirochaetes (**Figure 3B** and **Supplementary Table 3**). We saw significant, meaningful changes in the *ad libitum* (AL) animals, and more significantly in the high fat (HF) animals, as compared to our limit fed (LF) pigs starting at 10 Weeks post-dietary intervention (PD). Specifically in our *ad libitum* group, we observed a decrease in the phylum Bacteroidetes at 10 weeks (p= 0.03) and a decrease in the phylum Firmicutes at 14 weeks (p= 0.01), as compared to our limit fed animals. More significant changes were observed in our high fat group. Specifically, we observed again a decrease in Bacteroidetes starting at 10 weeks (p= 0.0009), that remained in lower abundance for the duration of the experiment as compared to our limit fed group. We observed this trend within the phylum Firmicutes as well. We saw an increase of Proteobacteria (p= 0.002) and Spirochaetes (p= 0.02) only in our high fat animals, both becoming significant at 10 weeks PD as compared to our limit fed group. Though all groups displayed an increase in Spirochaetes over time, we observed that this phylum was elevated in our high fat animals for the duration of the experiment. Overall, we observed meaningful shifts in microbial composition over time in our 3 groups starting at 10 weeks, with the most significant changes occurring in our high fat animals.

To more specifically identify bacteria undergoing the greatest changes in abundance, we evaluated the relative abundance at the taxonomic level of species in the growing pigs. Though some expected individual variation was observed, the microbiome of our growing pigs in all treatment groups were remarkably resilient towards dietary intervention. However, we did observe significant changes in bacterial species within the high fat group that were consistent with the composition of our *ad libitum* cohort in the first study (**Figure 3C** and **Supplementary Table 4**). For example, we observed an overall increasing trend in multiple *Treponema spp*, such as *Treponema porcinum* (p= 0.03 LF-HF) and *Treponema succinifaciens* (p= 4.60^-5 AL-HF), becoming significant at 10 weeks, and continuing throughout the duration of the experiment (*T. porcinum* [p= 0.04 LF-HF]). Additionally, we observed multiple *Prevotella* spp. change in abundance in response to the high fat diet. For example, *Prevotella sp P5-92* was increased in our high fat animals as compared to both our limit fed and *ad libitum* (p= 0.048) piglets, and became increasingly significant over time up to 14 weeks (p= 1.01^-6). Interestingly, *Prevotella copri* decreased over time in response to a high fat diet. Starting at 1 week PD, *Prevotella copri* was depleted in the high fat fed piglets. Finally, as in our endpoint *ad libitum* model, the high fat fed piglets displayed an overall decrease in *Lactobacillus amylovorus* as compared to the limit fed group, becoming significant at 18 weeks PD (p= 0.038). The high fat fed group also displayed changes in microbial abundances that were not detected in our initial study. For example, the high fat fed piglets underwent a depletion of *Streptococcus hydrointestinalis* starting at 10 weeks (p= 0.03 LF-HF; p= 7.17^ -6 AL-HF) which remained almost undetectable for the duration of the experiment. This trend was observed to a lesser extent in our limit fed piglets as compared to our *ad libitum* piglets starting at 10 week PD as well (p= 0.046). Overall, we observed multiple changes within our high fat piglets that closely resembled the microbial composition seen in our fully developed obese pigs. As in our preliminary study, bacterial taxonomic classification at the family and genus level is shown in **Supplementary Figure 5**, and supports the changes in the microbiome due to diet perturbation described above.

### The Microbiota Composition Was Altered in Mangalica Pigs Fed A High Fat Diet, Reflecting an Obesity-Associated Profile

**Figure 4:**
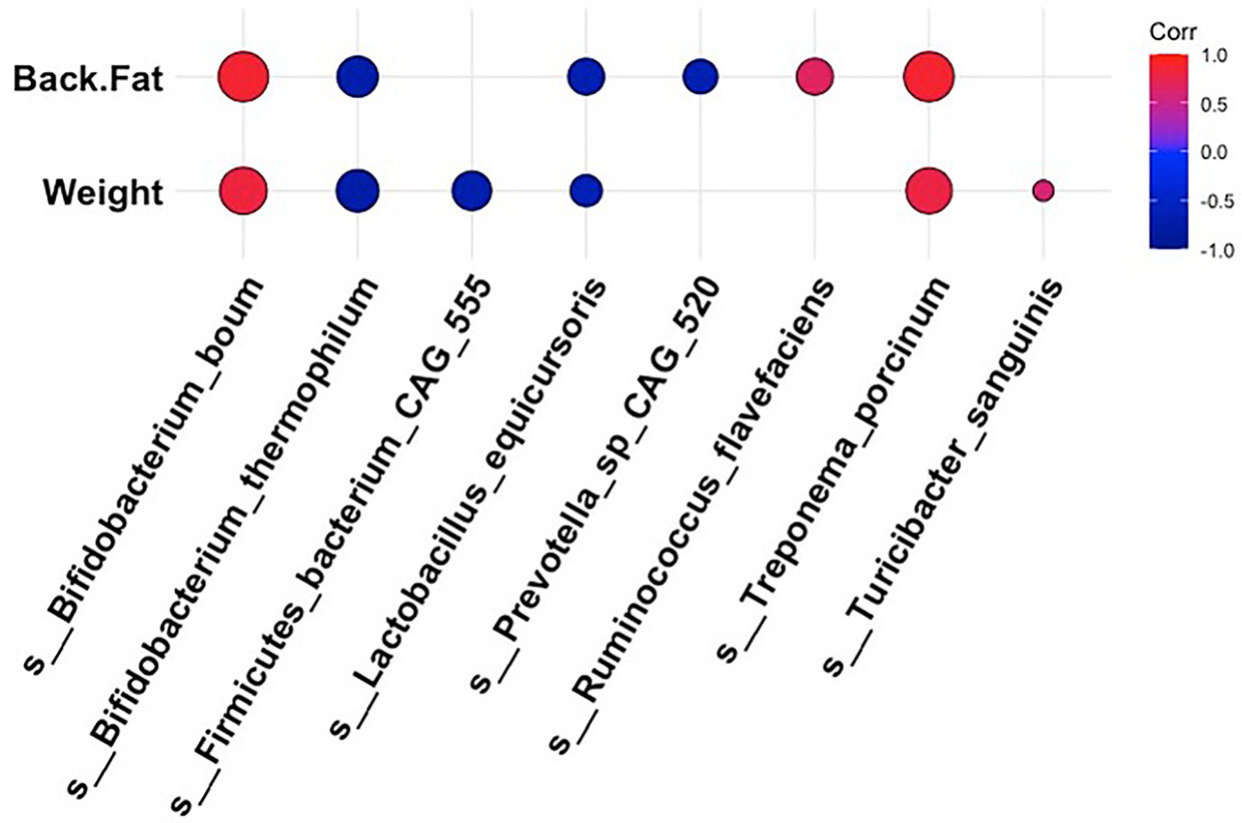
Correlations between bacterial species and metabolic phenotype in Mangalica pigs following 18 Weeks of dietary exposure. Pearson’s correlation plot of bacterial species and phenotypic data for Mangalica pigs fed a limit fed, *ad libitum*, or *ad libitum* + high fat diet. Data shown is after 18 weeks of dietary exposure. Statistical significance was determined for all pairwise comparisons. Positive values (red circles) indicate positive correlation coefficients above 0.6, and negative values (blue circles) indicate inverse correlation coefficients below -0.6. The size and shading of the circles indicate the magnitude of the correlation, where larger circles indicate a stronger correlation than smaller circles. Correlation coefficient values outside of |0.6| are not included in this plot.

To obtain a more holistic picture of how fluctuations in the intestinal microbiota related to the pig physiological phenotype, we analyzed the correlations between specific bacterial taxa and metabolic measurements at 18 weeks post dietary intervention (PD). Interestingly, the bacterial species that were more abundant within the intestinal microbiota of the high fat-fed animals also demonstrated a positive relationship with adipose accumulation and weight gain (**Figure 4**). In contrast, microbial constituents that were seen to dominate the intestinal microbiota profile of our limit fed group revealed an inverse relationship to adipose accumulation and weight gain (**Figure 4**). For example, we observed strong positive correlations between *Treponema porcinum* and *Bifidobacterium boum* with back fat and weight. This would indicate that an increase in weight due to increased adiposity is accompanied by a higher abundance of these intestinal microbiota constituents. This is consistent with elevated representation of these species, or species belonging to the same genus, within the *ad libitum* intestinal microbiota profile in the preliminary study. On the other hand, *Bifidobacterium thermophilum* and *Lactobacillus equicursoris* were both negatively correlated with back fat thickness and weight gain. *Lactobacillus* species were seen to be depleted in both our growing piglets and our fully developed obese pigs, which would indicate they are associated with leanness (**Figure 4**). Other species were found to be either weakly positively (*Ruminococcus flavefaciens* and *Turicibacter sanguinis*) or negatively (*Firmicutes bacterium CAG 555* and *Prevotella sp CAG 520*) correlated with our phenotypic markers. Taken together, the bacterial microbiota of developing Mangalica pigs was remarkably resilient to diet change. However, we did observe shifts within the high fat fed animals, resembling the *ad libitum* cohort in the initial study. Our data suggest that high dietary fat expedites changes within the bacterial constituents of the microbiota generating an obesity-associated microbiota profile.

### Bacteriophage Dynamics Were Altered by Age and Diet in Mangalica Pigs

**Figure 5:**
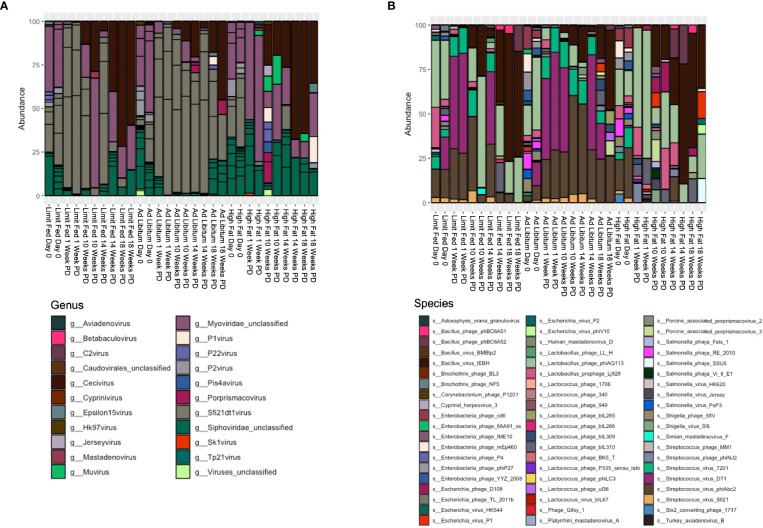
Changes in bacteriophage composition after dietary exposure in weaned Mangalica pigs. **(A)** Genus and **(B)** Species level viral composition derived from the order Caudovirales in Mangalica pigs fed either a limit fed, *ad libitum*, or *ad libitum* + high fat diet after 1, 10, 14, and 18 weeks of dietary exposure. Differential abundance statistical results outlined in **Supplementary Tables 5**, **6**.

Next, we wanted to define the viral composition within the intestinal microbiota of the Mangalica piglet in response to diet change. To do this, we calculated relative abundance of viruses at both the genus and species level. The gut was dominated by bacteriophages within the order Caudovirales, so our analysis was focused on looking specifically at these agents. As with the bacterial component of the intestinal microbiota, the *ad libitum* (AL) and limit fed (LF) piglets bacteriophage profiles were very similar in composition (**Figure 5A** and **Supplementary Tables 5**, **6**). In stark contrast to bacterial populations, we observed rapid and significant restructuring of bacteriophage composition in the piglet gut in response to the high fat diet (HF). These changes were visible first at the genus level (**Figure 5A** and **Supplementary Table 5**). Specifically, we saw a rapid and significant depletion of *Sfi21dt1* viruses in the high fat animals starting at 1 Week post dietary intervention (PD) (p= 3.99^-5 LF-HF; p= 2.09^-5 AL-HF), and this trend continued for the duration of the experiment (p= 0.02 AL-HF). Though not significant, we saw an overall increase in bacteriophage belonging to the genus *Myoviridae unclassified*, especially at later time points: 14 and 18 weeks PD.

To gain better resolution on phage dynamics in our growing piglets, we next characterized the relative abundance of phage populations at the level of species (**Figure 5B** and **Supplementary Table 6**). As expected, some bacteriophage followed the patterns of their host. For example, the virulent phage *Lactobacillus phage phiAQ113* and the temperate phage *Lactobacillus prophage Lj928*, which target *Lactobacillus* spp., followed a similar abundance pattern over time as their host. Additionally, consistent with the level of genus, we observed rapid changes in the bacteriophage populations at the taxonomic level of species in response to the high fat diet. Specifically, we saw rapid depletion of 4 of 6 annotated bacteriophage that target *Streptococcus* starting at 1 week. These 4 viruses belong to the genus *Sfi21DT1 viruses*, and include the temperate bacteriophage *Streptococcus virus Sfi21* (p= 0.0012 LF-HF; p= 0.0053 AL-HF), and the virulent bacteriophages *Streptococcus virus 7201* (p= 5.32^-5 LF-HF; p= 0.0004 AL-HF), *Streptococcus virus DT1* (p= 5.32^-6 LF-HF; 6.26^-5 AL-HF), and *Streptococcus phiABC2* (p=0.0015 LF-HF; p= 0.0053 AL-HF). These phage remained mostly undetectable in our high fat fed piglets for the duration of the experiment, while remaining fairly constant in our limit fed and *ad libitum* fed piglets. Interestingly, *Streptococcus* spp. were detectable in the high fat fed piglets at 1 week PD, yet by 10 weeks PD were significantly depleted. Collectively, numerous bacteriophage genera, specifically those who target *Streptococcus* spp., were reduced in abundance and their putative hosts also became depleted, yet more slowly.

**Figure 6:**
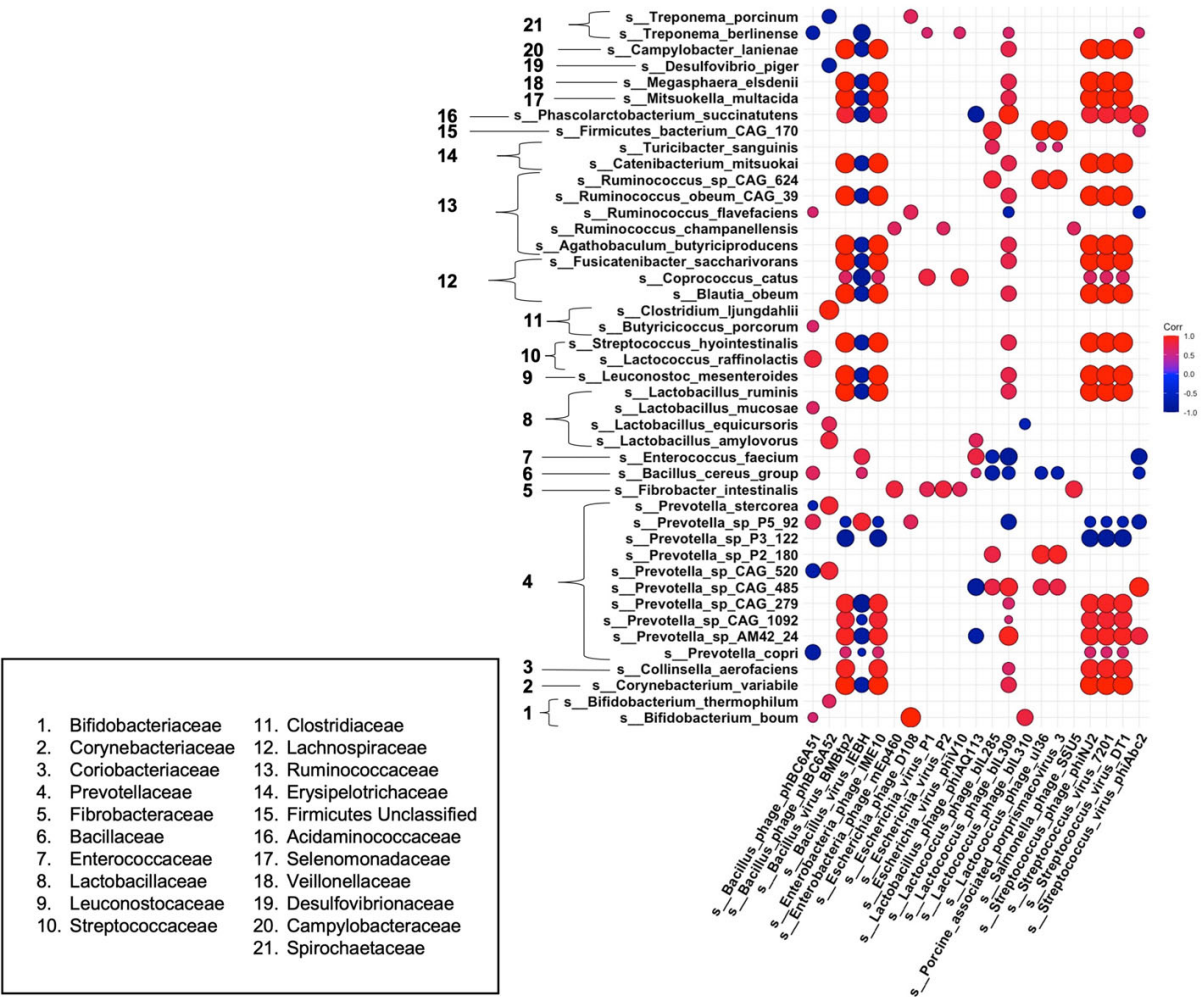
Correlations between bacteria and bacteriophage species at 18 weeks post dietary interventions. Pearson’s correlation plot of bacterial species *vs*. bacteriophage species after 18 weeks of dietary exposure. Statistical significance was determined for all pairwise comparisons. Positive values (red circles) indicate positive correlation coefficients above 0.6, and negative values (blue circles) indicate inverse correlation coefficients below -0.6. The size and shading of the circles indicate the magnitude of the correlation, where larger circles indicate a stronger correlation than smaller circles. Correlation coefficient values outside of |0.6| are not included in this plot.

Finally, to gain a better understanding of phage-host dynamics in our growing pigs, we analyzed the correlative relationships between bacteria and bacteriophage species at 18 weeks post dietary intervention (PD) (**Figure 6**). Strong positive correlative patterns were observed between many bacteria and phage species. Of note, many strong positive correlations were detected between a wide variety of bacterial species and *Streptococcus* – targeting phage, such as the temperate phage *Streptococcus phage phiNJ2*, and virulent phages *Streptococcus virus 7201* and *Streptococcus virus DT1.* This strong, positive pattern was also observed with the temperate phages *Bacillus virus BMBtp2* and *Lactococcus phage biL309*. Conversely, the *Bacillus* – targeting temperate phage*, Bacillus virus IEBH* was negatively correlated with a wide variety of bacterial species. Overall, this trend was observed in bacterial species that were not affected by diet perturbation, and remained consistent between diet groups throughout our experiment. As in our differential abundance analysis (**Figures 3**, **5**), we noticed that many bacteriophage correlated positively with their putative hosts. We observed this trend across multiple bacteria/phage pairs, including both temperate and virulent relationships. For example, *Streptococcus hydrointestinalis* was positively correlated with *Streptococcus phage phiNJ2, Streptococcus virus 7201*, and *Streptococcus virus DT1*. Additionally, the virulent phage *Lactobacillus phage phiAQ113* was positively correlated with one of its putative hosts, *Lactobacillus amylovorus*.

Some bacterial species we noted as being significantly different in our high fat fed pigs correlated uniquely with various bacteriophage species. For example, *Prevotella P5-92* was seen to be significantly elevated within our high fat pigs. We observed a negative correlation of this bacterial species with multiple *Streptococcus*-targeting bacteriophage that were depleted within our high fat fed group. Interestingly, other members of the *Prevotella* genus, such as *Prevotella copri*, were depleted within our high fat group and were positively correlated with *Streptococcus* – targeting bacteriophage. Additionally, *Ruminococcus flavefaciens*, which was positively correlated with back fat thickness and body weight (**Figure 4**), was weakly negatively correlated with the *Streptococcus* – targeting phage *Streptococcus virus phiAbc2.* Overall, we saw a mostly consistent pattern of correlation between bacteria and bacteriophage species, in which most bacteriophage followed the pattern of their host. However, we observed a deviation from this pattern in bacteria and phage that were significantly changed within our high fat animals, or ones that were positively correlated with phenotypic markers of our growing piglets.

Taken together, we found feeding different amounts of the same diet to young Mangalica pigs had only modest effects on the microbiota within the 18 weeks of this study. In contrast, when fed a diet supplemented with dietary lipids, we observed expedited changes within the gut microbiota that reflected the composition seen at the endpoint of obesity in our novel model, the Mangalica pig. Though significant changes were observed within the bacteriome of our high fat fed piglets, the repercussions of this diet were highlighted by the rapid restructuring of the virome. This also highlights the capability of bacteriophage to contribute to the restructuring of the bacteriome during the development of an obesogenic state, which is promoted by the presence of dietary lipids.

## Discussion

The prevalence of obesity is increasing worldwide in adults at an alarming rate given obesity associates with a multitude of comorbidities (CDC, 2020). Due to its proinflammatory, insulin resistant metabolic phenotype that develops concomitant with its extreme, early onset, morbidly obese body type, the Mangalica pig displays great potential to serve as a relevant animal model of obesity. Such a model of obesity could facilitate novel research aimed at either preventing obesity or uncoupling the obese phenotype from its underlying meta-inflammation (Roberts et al, under review). The aim of this study was to extend such observations by characterizing the intestinal microbiota in mature lean and obese Mangalica pigs and by determining the longitudinal effects of age and diet on the developing gut microbiota in juvenile Mangalica. These studies are the first to describe the overall development of the intestinal microbiome in the Mangalica pig and represent a key first step toward the development of this breed as a useful model to study mechanisms linking the intestinal microbiota and obesity.

Mature *ad libitum*-fed Mangalica pigs in the present study developed a striking degree of adiposity and presented similar characteristics to that of human obesity. These adult, obese pigs developed hyperglycemia and hyperinsulinemia suggesting that their obesity manifested a metabolic phenotype like that of humans suffering obesity-induced diabetes. This metabolic dysregulation was absent in leaner age-matched Mangalica pigs. The development of insulin resistance is a key link between obesity and downstream disease in humans and rodent models of obesity (González-Muniesa et al., 2017; Gomes et al., 2018). While these results confirm previous observations that the Mangalica breed serves as a novel swine model for obesity-induced metabolic disease. Importantly, they also provide key context for our characterization of the Mangalica gut microbiota. For instance, the changes observed within the gut microbiota in response to differences in body composition, age, and diet in the current study occurred amidst a backdrop of obesity-induced metabolic perturbations suggesting these changes in the microbiota were part of a sequence of events that are faithful to the etiology of human obesity.

The growth responses to dietary treatments in the present study were consistent with expectations for dietary manipulation of body composition in pigs. Importantly, limit fed pigs achieved a similar muscle mass as their *ad libitum* counterparts indicating the striking differences in their body weights were largely driven by differences in adiposity. This suggests that the differences observed in glucose and insulin levels were not confounded by differences in skeletal muscle mass but rather may have been a function of adiposity or due to changes in the gut microbiome as current models of obesity-induced insulin resistance maintain (Turnbaugh et al., 2009; Ley, 2010; Ridaura et al., 2013; Duvallet et al., 2017; González-Muniesa et al., 2017; Gomes et al., 2018). Interestingly, the distinct differences between the gut microbial composition of the limit fed (lean) and *ad libitum* (obese) pigs observed in the first study are consistent with a role for the gut microbiota. Such changes mirror the evolution of the human gut microbiota during development of human obesity as obese humans experience enrichment of bacteriophage concomitant with abundance changes in numerous bacterial phyla (Moreno-Gallego et al., 2019; Bikel et al., 2020; Gregory et al., 2020). This would be expected to both promote a more positive energy balance due to more efficient energy harvest from the diet as well as reflecting a more proinflammatory state (Yang et al., 2021).

Juvenile pigs that were limit fed, fed *ad libitum* or fed ad libitum + high fat diets during the second (longitudinal) study displayed the expected continuum in growth and body composition. While the gut microbiota was resilient to dietary treatment for pigs in the non-supplemented groups, juvenile pigs fed the high fat diet *ad libitum* displayed shifts in their microbiota that reflected a similar profile to that seen in our mature obese, *ad libitum*-fed pigs during the first study. It is unclear if this was due solely to the high fat component of the diet or if these changes in the microbiota were associated with changes in body composition as pigs fed the fat supplemented diets were also significantly fatter than pigs in the other dietary treatment groups. Nonetheless, results from the second (longitudinal) study suggest that it takes time for changes in intestinal microbiota within growing Mangalica pigs to reflect the lean or obese phenotype and this is likely due to age-related effects on adipose tissue development. The fattest pigs in the longitudinal study were still much leaner even at 18 weeks than the mature, obese pigs characterized in the first study (subcutaneous fat thickness of 46 mm *vs* 61mm respectively). The fact that fat supplementation expedited shifts in the gut microbiome might reflect the greater adiposity in this group.

To our knowledge, this study is the first to characterize the intestinal microbiota of the adult Mangalica pig in response to age and diet. The overall composition of the Mangalica gut microbiota was similar to that seen in human, mice, and other mammalian models (Turnbaugh et al., 2009; Ley, 2010; Ridaura et al., 2013). As in other pig models, we observed a domination of 4 main phyla: Bacteroidetes, Firmicutes, Proteobacteria, and Spirochaetes. The overall microbial consortia seen in our pigs was similar to the core porcine microbiota recently proposed (Holman et al., 2017). As in the meta-analysis performed by Holman et al, we noted large populations of *Lactobacillus* and *Prevotella* (Holman et al., 2017).

Within our various dietary groups, we observed unique differences in the intestinal microbiota of the *ad libitum*, obese adult pig that were supported by other fatty swine models (Pedersen et al., 2013; Curtasu et al., 2019). For example, we reported a significant increase in *Treponema* spp., which have been associated with fattiness in other swine models, such as the Jinhua pig and the Göttingen minipig (Pedersen et al., 2013; Yang et al., 2018). Interestingly, in another swine model of metabolic disease, the Ossabaw pig, *Treponema* spp. were associated with leanness (Panasevich et al., 2018). However, our study differs from Panasevich et al. in terms of diet. Potentially, diet composition is driving these opposing responses in *Treponema* spp. We also reported a decrease in some beneficial taxa, such as *Lactobacillus* spp., which is also consistent with other reports (Pedersen et al., 2013; Newman et al., 2021). Along with being a hallmark in obese human microbiomes, a decrease in beneficial *Lactobacillus* spp. has been observed in obese swine models such as the Ossabaw pig (Pedersen et al., 2013; Panasevich et al., 2018). In our study, we saw a decrease in *Lactobacillus amylovorus* in both our fully-developed obese pigs and our high-fat fed piglets. This specific species of *Lactobacillus* has been directly linked to the amelioration of obesity (Park et al., 2019).

It has been proposed that obese individuals, mice and humans alike, exhibit a higher proportion of Firmicutes to Bacteroidetes (Turnbaugh et al., 2006). However, previous reports show this is not always the case in human and swine studies (Pedersen et al., 2013; Finucane et al., 2014; Walters et al., 2014; Curtasu et al., 2019). The microbiome of our fully developed obese pig did not support this notion, in line with recent reports that conclude this ratio is not an indicator of host disease state (Finucane et al., 2014; Sze and Schloss, 2016; Duvallet et al., 2017). Host genetics plays a role in shaping the gut microbiota as well as contributing to the onset of obesity (Goodrich et al., 2014; Jou, 2014). Perhaps some of these findings reflect the unique characteristics of the Mangalica microbiota due to host genetics or environmental factors, as this idea has been recently supported in another swine model (Bergamaschi et al., 2020). Future studies could investigate how host genetics of swine breeds influences microbiota composition. Nonetheless, *ad libitum*-fed Mangalica pigs display an obese phenotype and the Mangalica intestinal microbiota responds to an obesogenic state in a somewhat unique manner relative to other models in the literature.

Juvenile Mangalica pigs were used to temporally describe how the microbiota responds to a limit fed, *ad libitum* or *ad libitum* + high fat (high fat) diet. When looking at the dissimilarity between the 3 groups over time, age was a more important factor in driving microbiota diversity and composition than diet. This observation is consistent with previous reports which have shown that a growing pig’s microbiota changes as it ages (Kim et al., 2011; Wang et al., 2019b). Regardless of diet, the constituents of the microbiota in both juvenile and mature Mangalica pigs were consistent with other reports describing the gut microbiota of piglets (Kim et al., 2011; Wang et al., 2019b). In the present study, we did not observe dramatic differences such as the loss of one more constituents of the gut microbiota. Rather, more modest changes in relative constituent abundances were present. This is to be expected, as all groups were fed the same diet and differed only in the amount of feed given, aside from the supplementation of high fat group. Interestingly, the largest changes in the juvenile gut microbiota occurred within our high fat group, indicating that macronutrient content might be more important than differences in caloric intake alone. Within our high fat samples, we reported changes in *Treponema* spp. and *Prevotella* spp. to be the most notable. *Prevotella* spp. have been reported to be associated with both lean and obese individuals, depending on the study (Precup and Vodnar, 2019). Further, recent reports by Ley et al. highlighted the genetic diversity within the genus *Prevotella*, indicating that the complete function of bacteria within *Prevotella* and their function in the gut microbiota might not yet be fully understood (Ley, 2016). This may explain why we observed opposing shifts in microbial abundance within the genus *Prevotella*. Additionally, the presence of *Prevotella*, regardless of abundance, may be linked to its ability to metabolize complex carbohydrates, which were present in all respective diets (de Filippo et al., 2010; Wu et al., 2011). Taken together, however, these data indicate that the juvenile pig’s gut microbiota is remarkably resilient to diet change.

Using shotgun metagenomic sequencing, we were able to describe populations in the gut microbiota other than bacteria, namely bacteriophage. Bacteriophage have recently been implicated as important modulators of the gut microbiota in health and disease (Howe et al., 2016; Manrique et al., 2016; Gogokhia et al., 2019; Hsu et al., 2019; Lin et al., 2019). We found that unlike the bacteriome, the virome was much more sensitive to diet change. In the high fat fed juvenile pigs, there was a rapid depletion of virulent *Streptococcus*-targeting viruses – specifically viruses that belonged to the genus *Sfi21dt1* viruses, both temperate and virulent (Hsu et al., 2019) Interestingly, we observed that *Streptococcus* spp. were not significantly depleted until 10 weeks post dietary intervention. Possible explanations for these seemingly contradictory trends include development of phage resistance by host bacteria or increased sensitivity of the phage to the gut environment. The notion that bacteriophage contribute to the restructuring of the bacterial community within the gut microbiota has been hypothesized before (Hsu et al., 2019). Our study provides support for this idea, as changes within the virome preceded that of the bacteriome. We have also provided evidence for this in a previous study using a mouse model of obesity (Higgins et al., 2021).

Though we annotated bacteriophage populations within our fully-developed obese pigs, variation between individuals was very high and we were not able to discern specific trends that corresponded with either fattiness or leanness. This was not completely unexpected however, as the virome has been reported to be one of the most variable parts of the gut microbiota (Moreno-Gallego et al., 2019). Moreno-Gallego et al. observed variability within the virome of monozygotic twins as the individuals aged, citing environmental variables as one possible explanation (Moreno-Gallego et al., 2019). In the present study, the 2 cohorts of pigs were housed in different environments, had different maternal lineages, and were different ages; all of which can impact the virome and gut microbiome in general. In addition to this, these pigs had been fed an *ad libitum* or a limit fed diet for a longer period of time as compared to our piglets. This gave time for increase adiposity and metabolic symptoms resulting from obesity. Perhaps the presence of these two factors contributed to the variability seen between the viromes of fully developed, obesogenic Mangalica pigs and growing Mangalica piglets.

One explanation for elevated bacteriophage abundance following dietary change could be that viruses replicate at a much faster rate than bacteria. Bacteria typically produce one daughter cell per replication cycle where one bacteriophage can give rise to hundreds of new virions within one host cell per replication cycle. Additionally, bacteriophage require less resources for production of progeny than bacteria. When the opportunity arises, such as a bloom in target bacteria following changes in nutrient availability, bacteriophage can benefit from the increase in viable host bacteria. In this way, a small bloom of bacteria could give rise to a rapid bloom of bacteriophage that target this host. Bacterial abundance levels could appear reduced or stagnant as bacteriophage progeny are infecting new daughter bacterial cells. Taken together, these data highlight the duality of the piglet microbiota. On one hand, the bacteriome was generally resilient to dietary change, while bacteriophage community rapidly restructured in the presence of dietary lipids. With recent reports indicating that bacteriophage can alter not only the abundance of bacterial constituents in the microbiota, but also metabolites they produce, it has become clearer that bacteriophage need further investigation as to their contribution to the overall structure and function of the gut microbiota (Hsu et al., 2019).

Though our study extensively characterized the metabolic and microbial characteristics of a novel swine model of obesity, it does not come without its limitations. Among these limiting factors were numbers of animals per group, translatability to humans, the type of fat administered in the high fat diet, and the DNA isolation method used. First, because of the scarce availability and the expense of care for this pig model, we were only able to include a limited number of animals in the study. In terms of microbial assemblages, there are also nuanced differences between a ‘typical’ human and pig microbiome, one of them being the presence of Spirochaetes, specifically *Treponema* spp. in the pigs. However, these microbes have been found in the gut microbiome of rural native individuals, who consume a diet high in plant polysaccharides, which is similar in nutritional composition to the diet administered here (Angelakis et al., 2019). Additionally, in future studies we will utilize a more common dietary fat found in human (western) diets rather than the soybean oil which is a more common additive to porcine diets (Wolp et al., 2012; Braundmeier-Fleming et al., 2020). Finally, the DNA extraction method used tends to favor bacteria-associated bacteriophage. Therefore, free phage genomes are likely depleted. Thus, differences in free phage might have been overlooked with this method, potentially obscuring differences in bacteriophage populations seen between samples in our study. Future studies utilizing this model should therefore include a larger sample size, different high fat additives and a DNA isolation method to enrich free phage to confirm the Manglica pig as a valid model to study human obesity.

In conclusion, these studies provide insight into how the swine intestinal microbiota responds to dietary changes and age. Pigs have been proposed as a more clinically relevant mammalian model to study human obesity compared to rodent models. These studies are the first to describe the progression of the intestinal microbiome composition in the Mangalica pig and are the first to provide evidence that changes in body composition and dietary conditions are associated with alterations in the microbiome of this novel porcine model of obesity.

## Data Availability Statement

The datasets presented in this study can be found in online repositories. The names of the repository/repositories and accession number(s) can be found in the article/**Supplementary Material**.

## Ethics Statement

The animal study was reviewed and approved by Auburn University Animal Care and Use Committee.

## Author Contributions

TB and EH conceived and designed the experiments. HH, KH, MR, JB, HM, TB, and EH performed the experiments. HH and KH developed the bioinformatic pipeline and analyzed the data. HH, TB, and EH prepared the manuscript. TB and EH supervised the study. All authors contributed to the article and approved the submitted version.

## Funding

This research was supported by the Alabama Agricultural Experiment Station and the Hatch program of the National Institute of Food and Agriculture, U.S. Department of Agriculture.

## Conflict of Interest

The authors declare that the research was conducted in the absence of any commercial or financial relationships that could be construed as a potential conflict of interest.

## Publisher’s Note

All claims expressed in this article are solely those of the authors and do not necessarily represent those of their affiliated organizations, or those of the publisher, the editors and the reviewers. Any product that may be evaluated in this article, or claim that may be made by its manufacturer, is not guaranteed or endorsed by the publisher.

## Supplementary Material

Supplementary Figure 1 Alpha Diversity of the intestinal microbiome of limit fed and *ad libitum* Mangalica Pigs. Rarefaction curves depicting the number of species compared to the number of reads in each sample. The total species count and total read count were used to generate rarefaction curves.

Supplementary Figure 2 Additional characterization of bacterial constituents of the intestinal microbiome in *ad libitum* and limit fed Mangalica pigs. Bar plots displaying relative abundance of bacteria at the **(A)** Family and **(B)** Genus level, in Mangalica pigs on either a limit fed or *ad libitum* feeding.

Supplementary Figure 3 Characterization of viral constituents of the intestinal microbiome in *ad libitum* and limit fed Mangalica pigs. Bar plots displaying relative abundance of viruses at **(A)** Genus and **(B)** Species level, in Mangalica pigs on either a limit fed or *ad libitum* feeding. Differential abundance statistical results outlined in **Supplementary Table 2**.

Supplementary Figure 4 Estimating Alpha Diversity over time after Dietary exposure in weaned Mangalica Pigs. Rarefaction curves were generated to determine the overall alpha diversity of each sample by comparing the total number of reads and total number of species within each sample at **(A)** Time 0, **(B)** 1 Week, **(C)** 10 Weeks, **(D)** 14 Weeks, and **(E)** 18 Weeks post-diet exposure.

Supplementary Figure 5 Additional characterization of the longitudinal impact on bacterial species in the intestinal microbiome following dietary exposure in weaned Mangalica pigs. A) Family, and B) Genus level bacterial composition in Mangalica pigs fed either a limit fed, *ad libitum*, or *ad libitum +* high fat diet prior to and after 1, 10, 14, and 18 weeks of dietary exposure.
