# Supplementary figures and images for: Longitudinal Analysis of the Intestinal Microbiota in the Obese Mangalica Pig Reveals Alterations in Bacteria and Bacteriophage Populations Associated With Changes in Body Composition and Diet
Source: Front Cell Infect Microbiol. 2021 Oct 19;11:698657. doi: 10.3389/fcimb.2021.698657 (PMC8560744; doi:10.3389/fcimb.2021.698657)

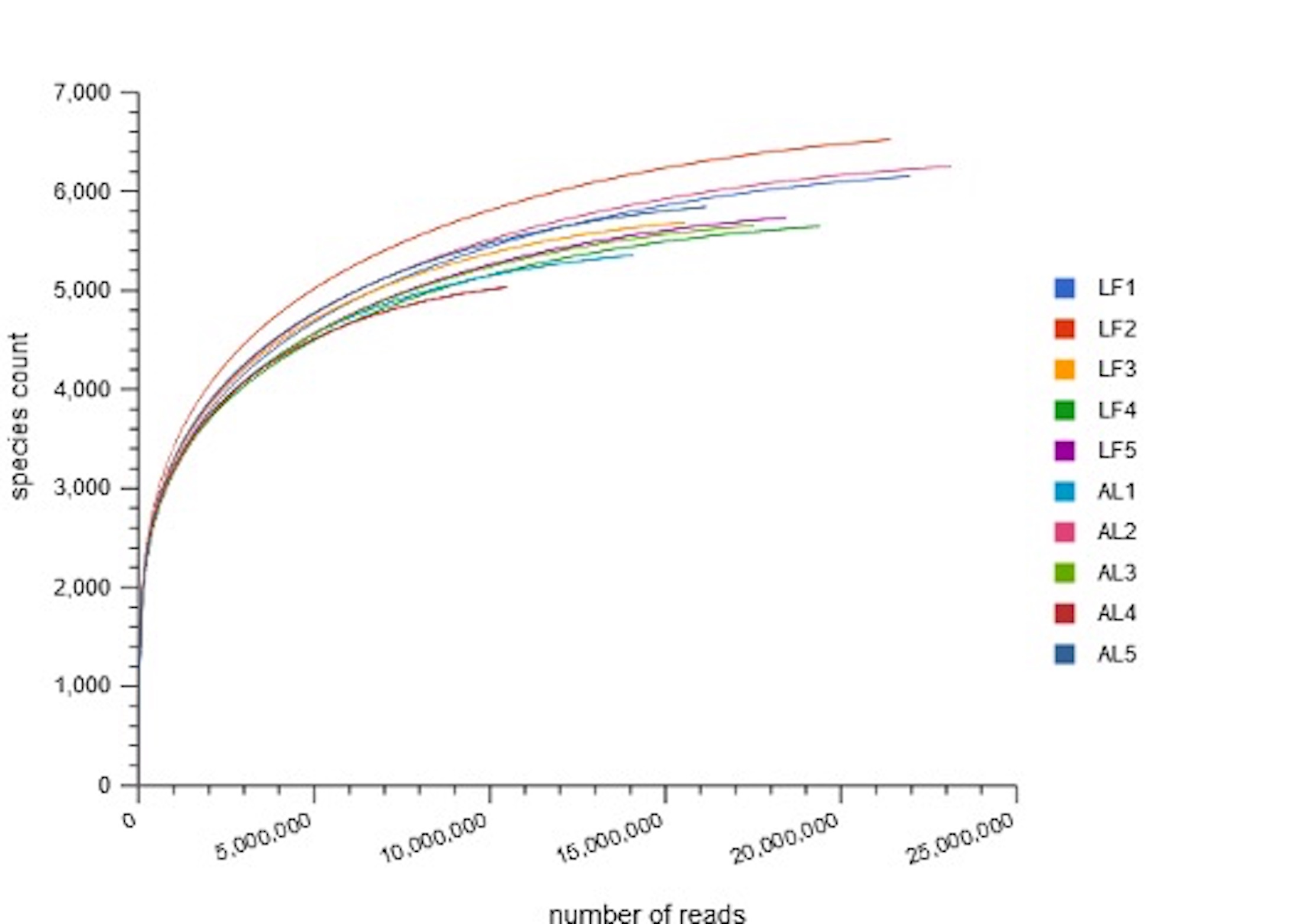

Supplement: Supplementary Figure 1 — Alpha Diversity of the intestinal microbiome of limit fed and ad libitum Mangalica Pigs. Rarefaction curves depicting the number of species compared to the number of reads in each sample. The total species count and total read count were used to generate rarefaction curves. [file Image_1.jpeg]

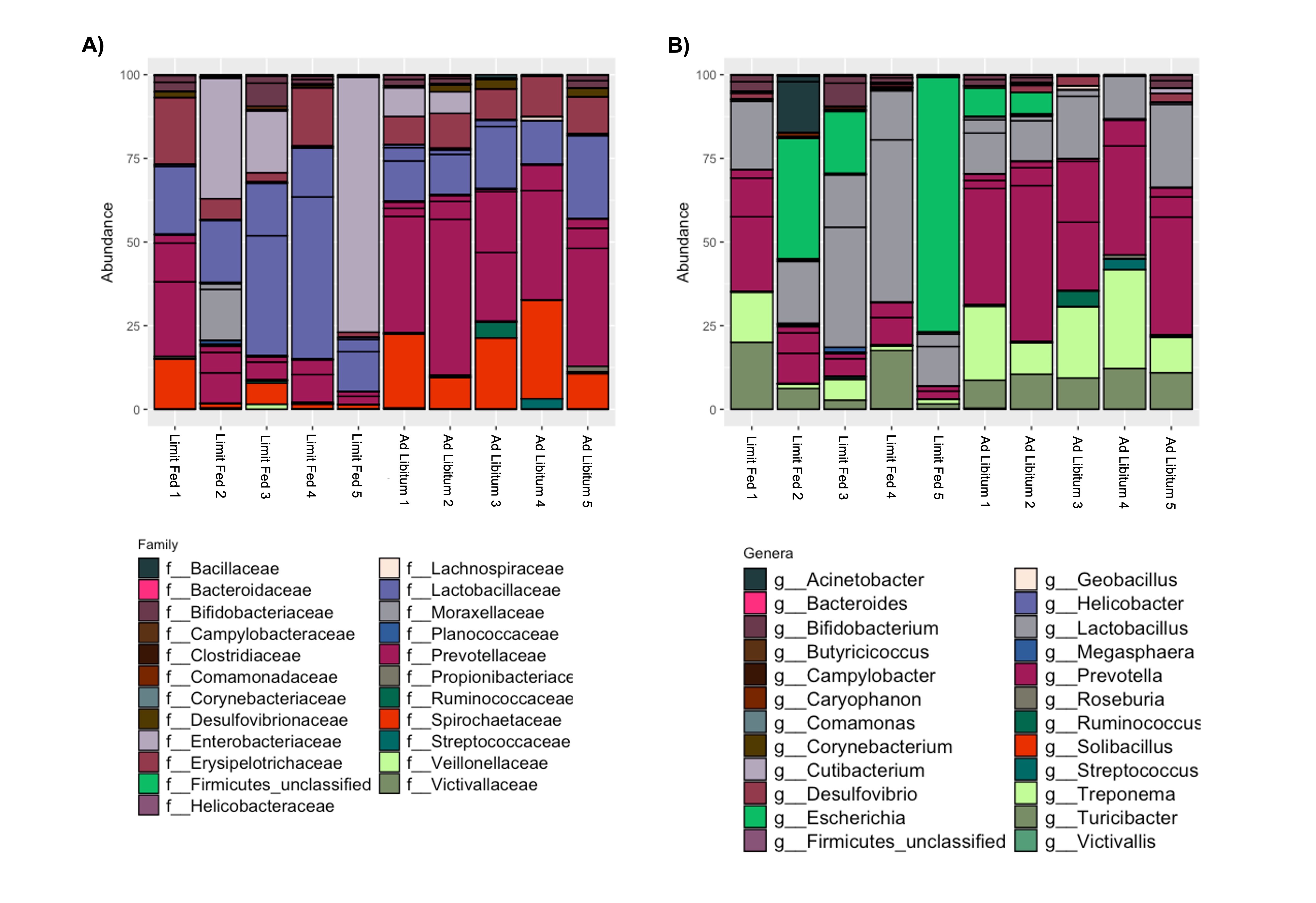

Supplement: Supplementary Figure 2 — Additional characterization of bacterial constituents of the intestinal microbiome in ad libitum and limit fed Mangalica pigs. Bar plots displaying relative abundance of bacteria at the (A) Family and (B) Genus level, in Mangalica pigs on either a limit fed or ad libitum feeding. [file Image_2.jpeg]

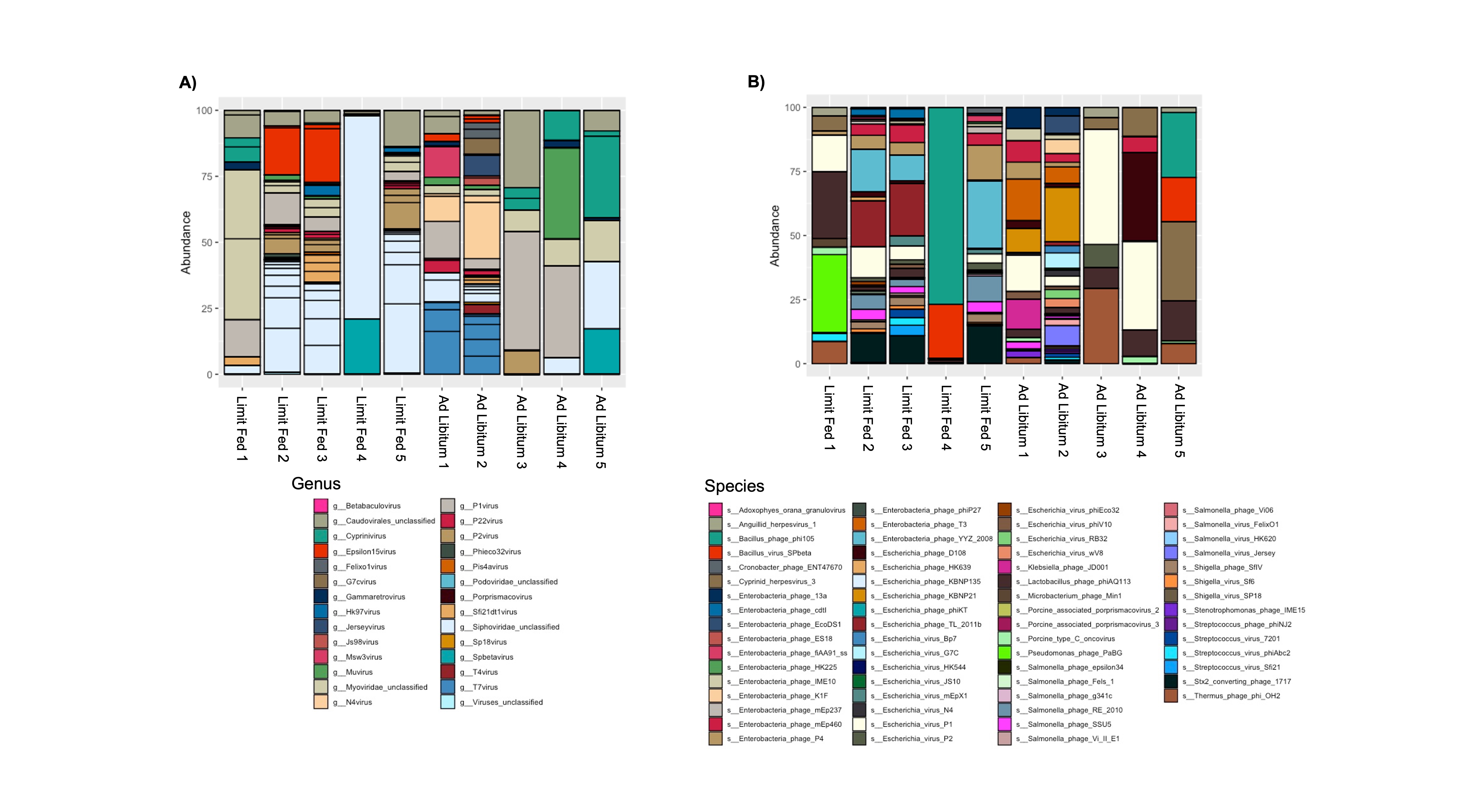

Supplement: Supplementary Figure 3 — Characterization of viral constituents of the intestinal microbiome in ad libitum and limit fed Mangalica pigs. Bar plots displaying relative abundance of viruses at (A) Genus and (B) Species level, in Mangalica pigs on either a limit fed or ad libitum feeding. Differential abundance statistical results outlined in Supplementary Table 2 . [file Image_3.jpeg]

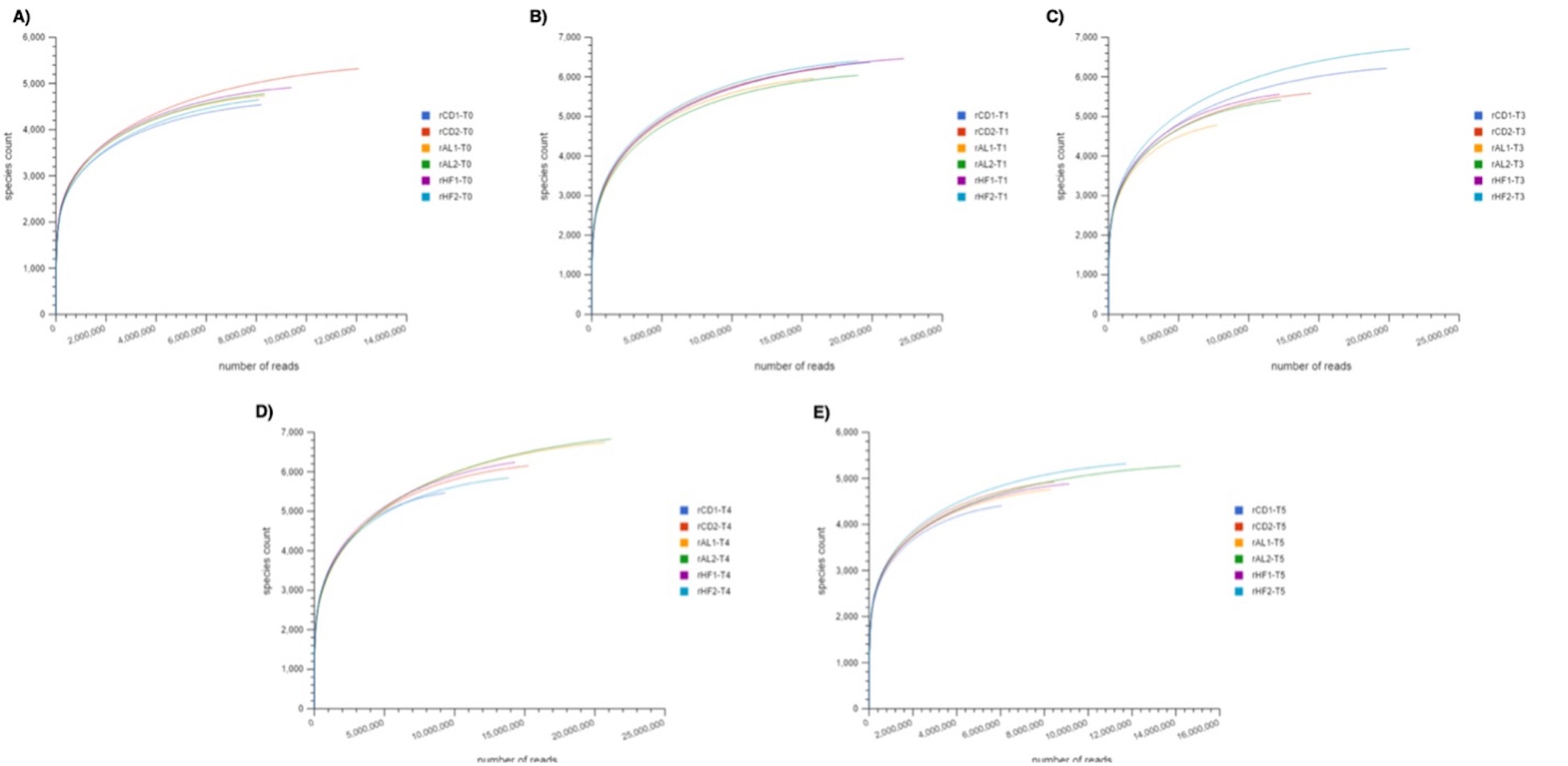

Supplement: Supplementary Figure 4 — Estimating Alpha Diversity over time after Dietary exposure in weaned Mangalica Pigs. Rarefaction curves were generated to determine the overall alpha diversity of each sample by comparing the total number of reads and total number of species within each sample at (A) Time 0, (B) 1 Week, (C) 10 Weeks, (D) 14 Weeks, and (E) 18 Weeks post-diet exposure. [file Image_4.jpeg]

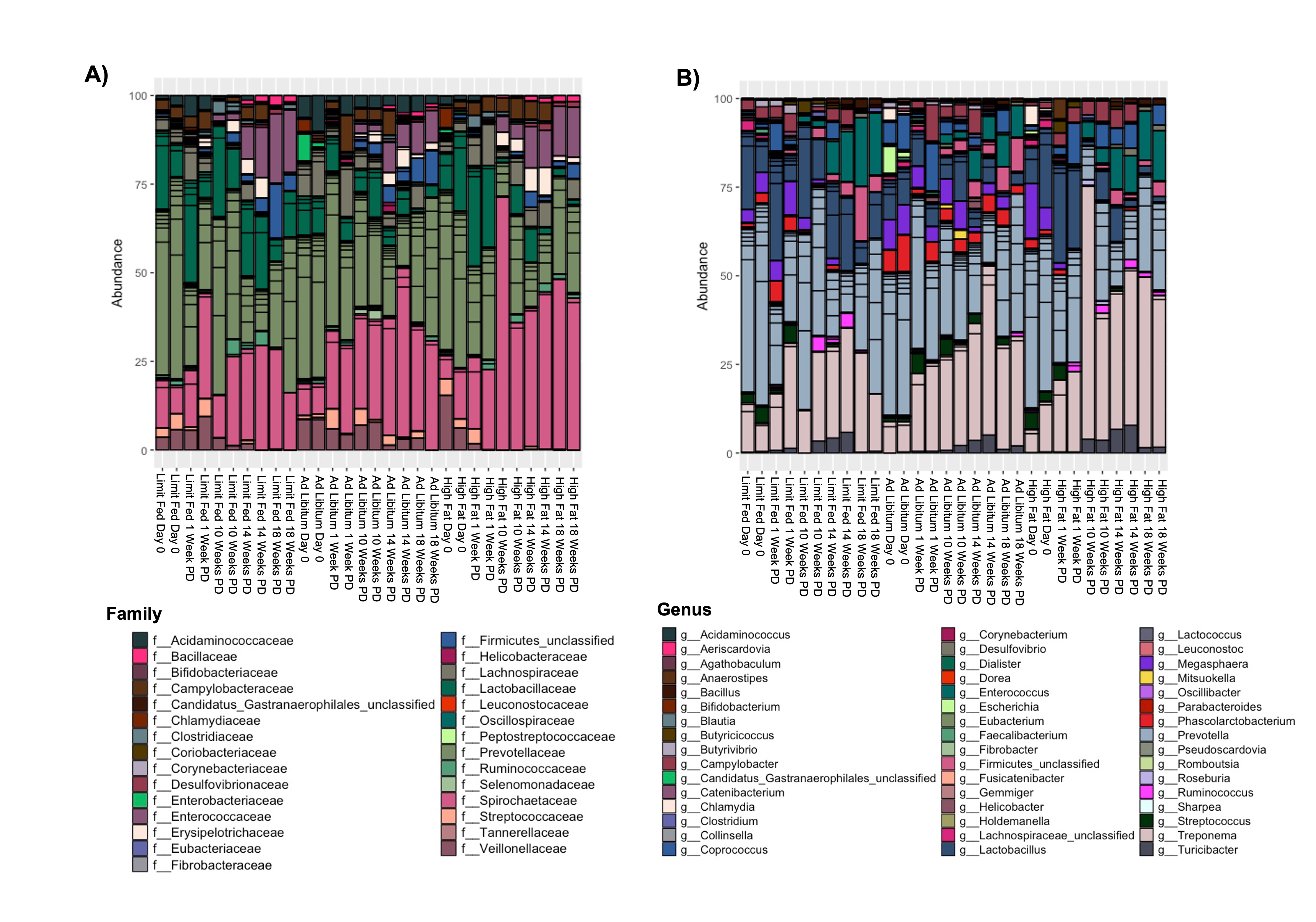

Supplement: Supplementary Figure 5 — Additional characterization of the longitudinal impact on bacterial species in the intestinal microbiome following dietary exposure in weaned Mangalica pigs. A) Family, and B) Genus level bacterial composition in Mangalica pigs fed either a limit fed, ad libitum, or ad libitum + high fat diet prior to and after 1, 10, 14, and 18 weeks of dietary exposure. [file Image_5.jpeg]
